# Protecting the mental health of youth

**DOI:** 10.1016/j.lanepe.2021.100306

**Published:** 2022-01-10

**Authors:** The Lancet Regional Health– Europe

Anxiety and depression are among the top five causes of ill health burden among youth population (age 15–29 years). Youth is the most formative period of life, and for more than half of adults with mental health disorders, first onset occurs during or before adolescence. Social isolation, disrupted education, and diminished job opportunities during the ongoing COVID-19 pandemic have exacerbated the disease burden for mental illness among youth. According to estimates from the State of the World's Children 2021 report by UNICEF, mental health disorders, including depression, anxiety, eating disorders, and attention-deficit hyperactivity disorder, are now present in 16.3% of European youth aged 10–19 years, an estimate that is higher than the global average of 13.2% for the same age group. To support youth population, the European Union (EU) has designated 2022 the ‘European Year of Youth’ to accelerate progress in areas previously defined as Youth Goals, including mental health and wellbeing, equality and inclusion, and sustainability.

The European Year of Youth 2022 is framed explicitly as an opportunity to accelerate the existing EU Youth Strategy (2019–27). The 2021 EU Youth Strategy progress report shows that achievements to date have been limited since the pandemic has stalled many of the youth mobility programmes that provide young people opportunities abroad, and only a few mental health programmes are included. Among these mental health programmes is the Joint Action on Mental Health (ImpleMENTAL) to support youth suicide prevention in EU countries. Suicide remains the second leading cause of death in European youth (after road traffic injuries) and thus strengthening suicide prevention is necessary, however, such a specific focus diverts from more common issues such as anxiety and depression. A study of helpline data in several European countries during the pandemic showed that increases in helpline calls was predominantly due to feelings of anxiety and loneliness rather than suicidal thoughts.

In the 2018 report of the Youth Goals, young people expressed their desire for greater access to mental health promotion and prevention programmes within their current environments (eg, schools, universities, youth clubs). Instead of waiting to treat mental health problems, integrating prevention interventions in everyday settings can increase positive mental health and contribute to better health, social, and academic outcomes for young people, an approach known as ‘mental health promotion’. Promoting awareness of lifestyle or behavioural factors that influence the onset of illness is central to any health promotion approach.

Mental health promotion programmes in schools specifically aim to teach young people social and emotional skills such as self-awareness and resilience so that they can recognise mental health problems in themselves and their peers and react appropriately. For example, Ireland has made Social, Personal and Health Education (SPHE) a compulsory part of the national school curriculum with an aim to promote physical, mental, and emotional wellbeing. In a randomised trial, the school-based MindOut social and emotional learning programme for adolescents, delivered within the SPHE curriculum, significantly improved students' social and emotional skills while reducing symptoms of stress and depression. The inclusion of all youth (instead of only high-risk students) and education for parents and teachers are important aspects of comprehensive health promotion programmes.

In addition to universal interventions, targeted programmes are needed within the school setting to provide more tailored support for students experiencing mental ill health and mental health services are needed outside of the school environment for the highest-risk students. However, interventions designed before the pandemic might no longer fit current needs. Today, digital approaches might be necessary, but the evidence for digital health promotion programmes is still emerging and highlights the need to consider supportive implementation structures to enhance engagement and reduce high drop-out rates. Additionally, specific measures are needed to reach underserved populations who face substantial barriers to online care and disproportionately carry the pandemic burden.

To ensure these programmes are tailored to the youth of today, meaningful youth engagement is key. The United Nations Population Fund Youth against COVID-19 campaign shared materials with young people to create video content about mental health that can be used to connect with peers during the pandemic. Such peer-to-peer initiatives allow young people to contribute meaningfully and connect with those outside the conventional setting of schools, universities, or workplaces. Organisations can learn from this example, and instead of designing new top-down campaigns, choose to amplify existing youth-led initiatives or design platforms for youth peer-to-peer contacts.

The European Year of Youth 2022 presents a window of opportunity for countries and organisations to enhance health promotion initiatives and to focus on mitigation of mental health problems. Mental health promotion in the youth of today can contribute to a ‘building back better’ pandemic recovery by creating a resilient generation of future EU leaders.

